# Automatic Skull Stripping of Rat and Mouse Brain MRI Data Using U-Net

**DOI:** 10.3389/fnins.2020.568614

**Published:** 2020-10-07

**Authors:** Li-Ming Hsu, Shuai Wang, Paridhi Ranadive, Woomi Ban, Tzu-Hao Harry Chao, Sheng Song, Domenic Hayden Cerri, Lindsay R. Walton, Margaret A. Broadwater, Sung-Ho Lee, Dinggang Shen, Yen-Yu Ian Shih

**Affiliations:** ^1^Center for Animal Magnetic Resonance Imaging, The University of North Carolina at Chapel Hill, Chapel Hill, NC, United States; ^2^Biomedical Research Imaging Center, The University of North Carolina at Chapel Hill, Chapel Hill, NC, United States; ^3^Department of Neurology, The University of North Carolina at Chapel Hill, Chapel Hill, NC, United States; ^4^Department of Radiology, The University of North Carolina at Chapel Hill, Chapel Hill, NC, United States; ^5^Department of Brain and Cognitive Engineering, Korea University, Seoul, South Korea

**Keywords:** rat brain, mouse brain, MRI, U-net, segmentation, skull stripping, brain mask

## Abstract

Accurate removal of magnetic resonance imaging (MRI) signal outside the brain, a.k.a., skull stripping, is a key step in the brain image pre-processing pipelines. In rodents, this is mostly achieved by manually editing a brain mask, which is time-consuming and operator dependent. Automating this step is particularly challenging in rodents as compared to humans, because of differences in brain/scalp tissue geometry, image resolution with respect to brain-scalp distance, and tissue contrast around the skull. In this study, we proposed a deep-learning-based framework, U-Net, to automatically identify the rodent brain boundaries in MR images. The U-Net method is robust against inter-subject variability and eliminates operator dependence. To benchmark the efficiency of this method, we trained and validated our model using both in-house collected and publicly available datasets. In comparison to current state-of-the-art methods, our approach achieved superior averaged Dice similarity coefficient to ground truth T2-weighted rapid acquisition with relaxation enhancement and T2^∗^-weighted echo planar imaging data in both rats and mice (all *p* < 0.05), demonstrating robust performance of our approach across various MRI protocols.

## Introduction

Magnetic resonance imaging (MRI) is a widely employed technique to study brain anatomy and function in preclinical rodent models (Mandino et al., 2020). To achieve individual subject data standardization and facilitate group level comparison, pre-processing must remove non-brain tissue, a.k.a. skull strip; without it, the automatic registration process would likely fail due to unwanted signal outside the brain. In many cases, skull stripping is achieved by manually drawing brain masks for each individual slice, making it a time-consuming and operator-dependent process. Ideally, an automatic skull stripping tool would streamline the pre-processing pipeline, avoid personnel bias, and significantly improve research efficiency, especially when handling large datasets (Babalola et al., 2009; Lu et al., 2010; Gaser et al., 2012; Feo and Giove, 2019). In human MRI research, several automatic brain extraction tools have been developed and widely utilized (Cox, 1996; Shattuck and Leahy, 2002; Leung et al., 2011; Doshi et al., 2013). However, these tools are not applicable to rodent applications because of differences in brain/scalp tissue geometry, image resolution with respect to brain-scalp distance, tissue contrast around the skull, and sometimes signal artifacts from surgical manipulations. Additionally, rodent brain MRI data is typically acquired at higher magnetic fields (mostly >7T) with higher radiofrequency (RF) coil inhomogeneity. The stronger susceptibility artifacts and field biases represent further challenges to the rodent skull stripping process.

To date, several attempts have been made to address rodent skull-segmentation (Pfefferbaum et al., 2004; Sharief et al., 2008; Bendazzoli et al., 2019; Feo and Giove, 2019; Lohmeier et al., 2019; Liu et al., 2020). To date, the most prominent tools for rodent MRI skull stripping are Pulse-Coupled Neural Network (PCNN)-based brain extraction proposed by Chou et al. (2011), Rapid Automatic Tissue Segmentation (RATS) pioneered by Oguz et al. (2014), and, and SHape descriptor selected External Regions after Morphologically filtering (SHERM) by Liu et al. (2020). Pulse-Coupled Neural Network is a biomimetic neural network initially developed for cat visual cortex segmentation (Kuntimad and Ranganath, 1999) that utilizes an iterative process to assign labels to adjacent pixels with similar intensity profiles. The RATS technique is built on mathematical morphology and LOGISMOS-based graph segmentation methods (Yin et al., 2010). While the RATS method has superior performance on T1-weighed images (T1w; Oguz et al., 2014), it is worth noting that T2-weighted images (T2w) and T2^∗^-weighted images (T2^∗^w) are also common choices in high-field brain function studies. The recently proposed SHape descriptor SHERM (Liu et al., 2020) method identifies a set of brain mask candidates, extracted from MRI images with multiple kernel sizes that matches the shape of the brain template. One common limitation of these brain segmentation methods is that the performance varies by brain size, shape, texture, and contrast, and therefore the technique needs to be optimized for each MRI protocol. Taken together, the development of a rodent skull stripping tool capable of performing on a variety of datatypes with accuracy and consistency is highly desirable.

Instead of using rules designed by users, learning-based methods acquire mapping functions from inbuilt feature engineering and classifiers, which would likely be more robust to various imaging modalities. Specifically, deep-learning-based methods combine feature engineering and classifiers into a uniform framework, and have achieved outstanding performance on many medical imaging identification tasks (Kleesiek et al., 2016; Havaei et al., 2017; Roy et al., 2018). Here we propose a novel model that adopts a fully convolutional deep-learning network, U-Net (Ronneberger et al., 2015; Yogananda et al., 2019), to perform dense feature extraction. The whole network is implemented using Keras (Chollet, 2015) with TensorFlow (Abadi et al., 2016) as the backend. We trained and tested the U-Net model for skull stripping performance using rat and mouse datasets that contained different imaging contrasts [i.e., T2w rapid acquisition with relaxation enhancement (T2w RARE) and T2^∗^w using echo planar imaging (T2w EPI)]. The performance of our proposed model was then compared with existing rodent skull stripping tools, including RATS, PCNN, and SHERM across different available datasets.

## Materials and Methods

### Dataset Descriptions

This study includes two separate datasets: an in-house collected dataset (CAMRI dataset) and an open source dataset (Online dataset) downloaded from http://openneuro.org. The CAMRI dataset consisted of 132 adult male rats of different strains [94 Sprague Dawley (SD), 22 Long-Evans (LE), and 16 Wistar: this dataset is available at https://doi.org/10.18112/openneuro.ds002870.v1.0.0] and 16 wild-type adult C57Bl/6J strain mice (the dataset is available at https://doi.org/10.18112/openneuro.ds002868.v1.0.0). For each animal, a T2w RARE and an T2^∗^w EPI were acquired. Among the 132 rats, 69 rats’ T2w RARE and T2^∗^w EPI resolutions were 0.1 mm × 0.1 mm × 1 mm and 0.32 mm × 0.32 mm × 1 mm, respectively, and the other 63 rats’ T2w RARE and T2^∗^w EPI resolutions were 0.2 mm isotropic and 0.4 mm isotropic, respectively. For the mice, the T2WI and T2^∗^w EPI resolutions were 0.16 mm isotropic and 0.32 mm isotropic, respectively. All CAMRI data were acquired on a Bruker 9.4T system. The Online dataset consisted of 24 rats and 36 mice. Specifically, T2w RARE of 24 female adult Wistar strain rats (Sirmpilatze et al., 2019),^1^ T2w RARE of 16 male and female B6.Cg-Tg(Fev-cre)1Esd/J mice (ePet-cre; RRID:IMSR_JAX:012712) (Grandjean et al., 2019),^2^ and T2^∗^w EPI images of 20 C57Bl/6J male and female mice (Grandjean et al., 2020).^3^ To train our U-Net model, we first established training dataset by randomly selecting 80% of the T2w RARE and T2^∗^w EPI images in the CAMRI rat data (78 SD, 15 LE, and 12 Wistar) as well as all CAMRI mouse data, leaving the remaining 20% of the rats as final performance testing dataset. In the training process, we further randomly selected 80% of the rat data from the training dataset (62 SD, 12 LE, and 10 Wistar) and included all mouse data for inner training. The remaining 20% of the rat data from the training dataset was used to validate the U-Net model. We repeated the training-validation process five times to avoid randomness in the data splitting. The U-Net model with the highest averaged validation accuracy was then used as the final model for testing.

To further illustrate the robustness and wide applicability of our proposed model in separate rat and mouse datasets, we tested our trained U-Net model on the Online dataset that was acquired from different scanners and with different imaging parameters.

### U-Net

**FIGURE 1:**
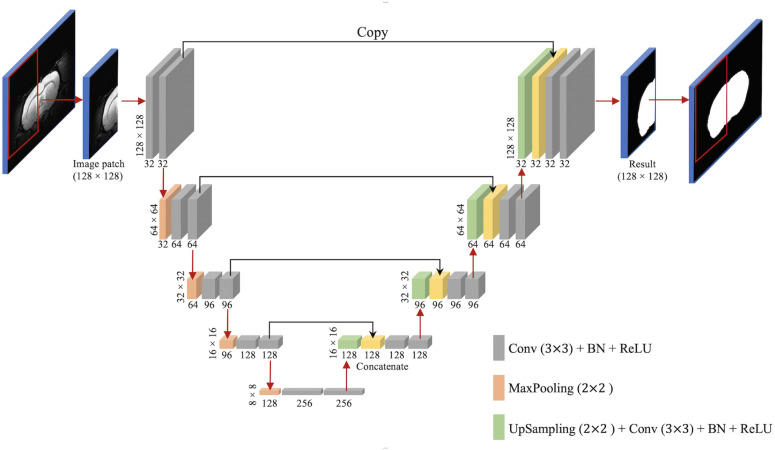
U-Net architecture. Boxes represent cross-sections of square feature maps. Individual map dimensions indicated on lower left, and number of channels indicated below dimensions. The leftmost map is a 128 × 128 normalized MRI image patched from the original MRI map, and the rightmost represents binary ring mask prediction. Red arrows represent operations, specified by the colored box, while black arrows represent copying skip connections.

We used U-Net (Ronneberger et al., 2015), a method with excellent performance in many medical image segmentation tasks (Ronneberger et al., 2015; Zhou et al., 2018; Alom et al., 2019; Yogananda et al., 2019; Wang et al., 2020b), to perform skull stripping on rodent brain MR images (Figure 1). In the contracting path, there are 32 feature maps in the first convolutional block, 64 in the second, then 96, 128, and 256 in the third, fourth, and fifth, respectively. Compared to the configuration described by Ronneberger et al. (2015), we replaced the cross-entropy loss function with the Dice coefficient loss (Wang et al., 2020a) to free the optimization process from a class-imbalance problem (Milletari et al., 2016).

In this study, since we include various rats and mice dataset (CAMRI and online dataset) with different image resolutions, we performed two different normalizations to improve the capabilities of the model: spatial normalization and intensity normalization. For spatial normalization, we resampled all images into the same spatial resolution at 0.1 mm × 0.1 mm slice-by-slice using nearest-neighbor interpolation. The nearest-neighbor interpolation was chosen to keep consistency in the processing pipeline because both brain-mask (binary) and brain image (grayscale) need to be resampled. Resampling was not performed across slices because we performed 2D U-Net slice-by-slice. For intensity normalization, we performed the min-max normalization for each image to range intensities from 0 to 1 and stored them as single precision (float-32). In U-Net training, the voxels belonging to the rat brain are labeled as 1 and other voxels (background) are labeled as 0. Our network was implemented using Keras (Chollet, 2015) with TensorFlow (Abadi et al., 2016) as the backend. The initial learning rate and batch size were 1e^–3^ and 16, respectively. We used Adam (Kingma and Ba, 2015) as the optimizer and clipped all parameter gradients to a maximum norm of 1. In training, we randomly cropped the 128 × 128 sized patches from all axial slices as the input. In the inference, the overlapped patches extracted from each axial slice were input into the trained model with a 16 × 16 × 1 stride. The overlapped predictions were averaged and then resampled back to the original resolution using nearest-neighbor interpolation for the final output.

### Evaluation Methods

To demonstrate the reliability of our proposed method, we compared our U-Net method with the most prominently used methods for rat brain segmentation: RATS (Oguz et al., 2014), PCNN (Chou et al., 2011), and SHERM (Liu et al., 2020). All images were bias-corrected for field inhomogeneities using Advanced Normalization Tools (ANTs).^4^ Since we included multiple datasets in this study, the parameters were chosen according to best parameters suggested in the publication to maintain consistency. For the RATS algorithm, the intensity threshold (T) was set to the average intensity in the entire image and the brain size values V_t_ was set to 1650 mm^3^ for the rat images and 380 mm^3^ for mouse images (Oguz et al., 2014). For the PCNN algorithm, the brain size range was set to 1000–3000 mm^3^ for rat images and 350–550 mm^3^ for mouse images (Chou et al., 2011). For SHERM, the brain size range was set to 500–1900 mm^3^ for rat images and 300–550 mm^3^ for mouse images (Liu et al., 2020). The default convexity threshold in SHERM, defined as the ratio between the volume of a region and that of its convex hull, was set to 0.85 to discard brain mask candidates. We adjusted the convexity threshold to 0.7 because brain mask candidate did not survive in half of the rodent images from CAMRI and online datasets, likely due to differences in raw data dimensions.

To quantitatively evaluate the segmentation performance of U-Net, RATS, PCNN, and SHERM, we estimated the similarity of the brain segmentation results generated by each method compared to manual drawing of brain masks by an anatomical expert according to the Paxinos and Watson rat atlas (Paxinos and Watson, 2014) and Konsman mouse atlas (Konsman, 2003). The manual segmentation was performed at the original MRI resolution before data resampling to 0.1 mm × 0.1 mm for U-Net training. To evaluate the reliability of the manual delineations (ground truth), we included two additional experts with profound knowledge of rodent brain anatomy and estimated the inter-rater accuracy compared to ground truth using 20 randomly selected rats (both T2w RARE and T2^∗^w EPI images). High reliability (accuracy > 0.95, Supplementary Figure 2) of the ground truth was found. Evaluations included: (1) volumetric overlap assessments via Dice, the similarity of two samples; (2) Jaccard, the similarity of two samples where Dice doesn’t satisfy the triangle inequality; (3) positive predictive value (PPV), the rate of true positives in prediction results; and (4) sensitivity (SEN), the rate of true positives in manual delineation; as well as (5) a surface distance assessment by Hausdorff distance, the distance of two samples. The following definitions were used for each: *Dice* = 2(|*A*∩*B*|)/(|*A*| + |*B*|), *Jaccard* = (|*A*∩*B*|)/(|*A*∪*B*|), *PPV* = (|*A*∩*B*|)/*B*, *SEN* = (|*A*∩*B*|)/*A*, and *Hausdorff* = *max*{*h*(*A*, *B*), *h*(*B*, *A*)} and *h*(*A*, *B*) = *max* { *min d*(*a*,*b*)} where *A* denotes the voxel *a* ∈ *A b* ∈ *B* set of the manually delineated volume, *B* denotes the voxel set of the predicted volume, and *d*(*a, b*) as the Euclidian distance between *a* and *b*. The Hausdorff distance was only estimated in-plane to avoid confounds from non-uniformly sampled data. The maximal Hausdorff distance (i.e., worst matching) across slices for each subject was then used for comparison. Superior performance was indicated by higher Dice, Jaccard, PPV, and SEN, and lower Hausdorff values. We also reported the computation time on a Linux-based [Red Hat Enterprise Linux Server release 7.4 (Maipo)] computing system (Intel E5-2680 v3 processor, 2.50 GHz, 256-GB RAM) for each method. The computation times reported do not include any preprocessing steps (i.e., signal normalization, image resampling, and bias correction). Paired t-tests were used for statistical comparisons between different algorithms, and two-sample t-tests were used to compare T2w RARE and T2^∗^w EPI images in each algorithm. The threshold for significance was set to the alpha level (*p* < 0.05).

## Results

**FIGURE 2:**
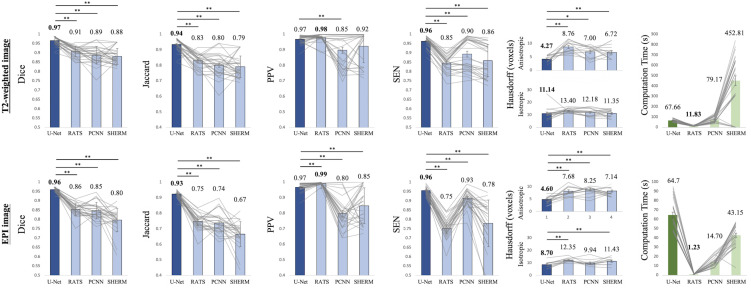
Segmentation performance for U-Net, RATS, PCNN, and SHERM on the T2w RARE **(upper row)** and T2*w EPI **(lower row)** images from CAMRI dataset. Average value is above each bar. Two-tailed paired t-tests were used for statistical comparison between U-Net with RATS, PCNN, and SHERM. Best performance results in bold (*p < 0.05 and **p < 0.01).

Figure 2 illustrates the performance of our trained U-Net algorithm compared to RATS (Oguz et al., 2014) and PCNN (Chou et al., 2011) for rat brain segmentation in the CAMRI dataset. In all measures, U-Net performed significantly better than the other two methods, except PPV was slightly inferior to RATS on the T2^∗^w EPI dataset. Notably, U-Net produced near-perfect results with all measures of volumetric overlap > 0.90. In contrast, the high PPV (0.98 on T2w RARE and 0.99 on T2^∗^w EPI) but low SEN (0.85 on T2w RARE and 0.75 on T2^∗^w EPI) from RATS indicates segmentation was underestimated, while the low PPV (0.85 on T2w RARE and 0.72 on T2^∗^w EPI) and high SEN (0.90 on T2w RARE and 0.93 on T2^∗^w EPI) in PCNN indicates segmentation was overestimated. The significantly lower Hausdorff distance in U-Net (4.27 on anisotropic T2w RARE and 4.60 on anisotropic T2^∗^w EPI) further indicates its best match segmentation. However, the U-Net algorithm had longer computation time than others using the same computational environment (67.66 s on T2w RARE and 64.70 s T2^∗^w EPI). In summary, the high accuracy (Dice > 0.95) of U-Net in training, validating (Supplementary Figure 1), and final performance testing demonstrates the reliability and consistency of our method.

There were no significant differences in segmentation performance between T2w RARE and T2^∗^w EPI with U-Net, but a significant decrease in performance was found with the other three algorithms (All *p* < 0.05, Figure 2). Specifically, the Dice, Jaccard, PPV, and SEN from RATS, the Dice, Jaccard, and PPV from PCNN, and the Dice, Jaccard, PPV, and SEN from SHERM were lower for T2^∗^w EPI than T2w RARE. The compromised performance in the T2^∗^w EPI image compared with T2w RARE indicates the challenges these three methods have with low resolution images.

**FIGURE 3:**
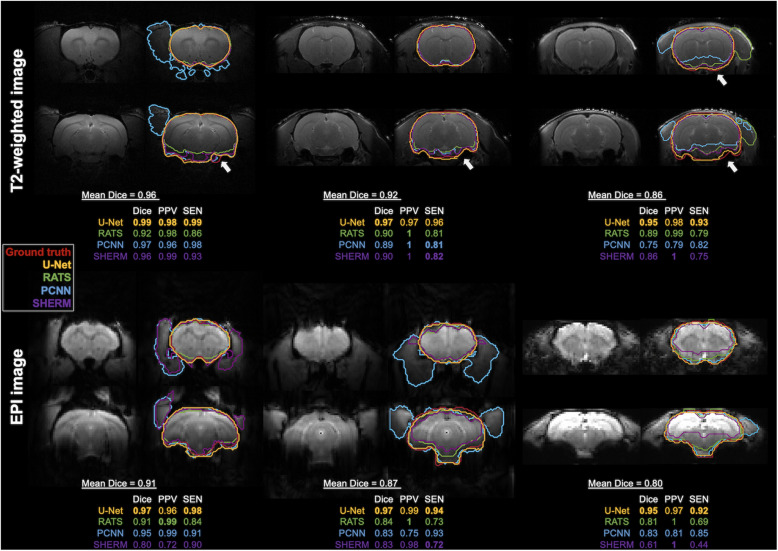
Best, median, and worst segmentation comparisons for T2w RARE and T2*w EPI images from CAMRI dataset. These rats were chosen as they had the highest, median, and lowest mean Dice score (listed below the brain map) averaged over the four methods (U-Net, RATS, PCNN, and SHERM). Posterior and inferior slices (arrowhead) are more susceptible to error in RATS, PCNN, and SHERM, whereas U-Net performs similarly to the ground truth.

Figure 3 illustrates the best, median, and worst cases on T2w RARE and T2^∗^w EPI from the CAMRI dataset using all four algorithms. These chosen rats had the highest, median, and lowest Dice score averages over the four methods. Note that in the worst case the RATS, PCNN, and SHERM algorithms failed to identify the brainstem, olfactory bulb, and inferior brain regions where the MRI signal was weaker. Supplementary Figure 3 illustrates more results for T2^∗^w EPI images. Importantly, U-Net could still achieve a satisfactory segmentation in the worst cases with Dice > 0.95 for both T2w RARE and T2^∗^w EPI. Compromised MRI signal intensity causes problems for RATS, PCNN, and SHERM algorithms, while U-Net still produces near-perfect results.

We included the Online dataset to illustrate the performance of our proposed algorithm on independent rat and mouse datasets. Table 1 indicates segmentation performance for rat T2w RARE. U-Net performed significantly better than RATS, PCNN, and SHERM on nearly all measures except PPV. Both T2w RARE (Table 2) and T2^∗^w EPI (Table 3) skull stripping in the mouse dataset were significantly improved in U-Net versus the other two methods except for PPV and Hausdorff distance. Overall, these results indicate that the proposed U-Net method is a highly competitive alternative to other existing skull stripping tools.

**TABLE 1:** Quantitative comparison of U-Net, RATS, PCNN, and SHERM for segmentations on rat T2w RARE from Online dataset.

Methods	Dice	Jaccard	PPV	SEN	Hausdorff (voxels)
U-Net	**0.94 (0.00)**	**0.88 (0.01)**	0.94 (0.00)	**0.98 (0.01)**	**6.81 (0.44)**
RATS	0.89 (0.02)	0.82 (0.03)	**0.95 (0.01)**	0.86 (0.02)	8.38 (0.40)
PCNN	0.85 (0.02)	0.75 (0.03)	0.84 (0.03)	0.88 (0.02)	9.16 (0.91)
SHERM	0.85 (0.02)	0.75 (0.02)	0.95 (0.01)	0.78 (0.03)	9.81 (0.88)
*p*-value (U-Net vs. RATS)	<0.05	<0.05	N.S.	<0.05	<0.005
*p*-value (U-Net vs. PCNN)	<0.001	<0.001	<0.005	<0.005	<0.05
*p*-value (U-Net vs. SHERM)	<0.001	<0.001	N.S.	<0.001	<0.005

*The *p*-value indicates the result of two-tailed paired *t*-test comparison (best performance results in bold).*

**TABLE 2:** Quantitative comparison of U-Net, RATS, PCNN, and SHERM for segmentations on mouse T2w RARE from Online dataset.

Methods	Dice	Jaccard	PPV	SEN	Hausdorff (voxels)
U-Net	**0.85 (0.01)**	**0.74 (0.01)**	0.74 (0.01)	**0.98 (0.00)**	5.23 (0.37)
RATS	0.82 (0.01)	0.70 (0.01)	**0.76 (0.01)**	0.91 (0.01)	**5.07 (0.31)**
PCNN	0.79 (0.00)	0.65 (0.01)	0.76 (0.01)	0.83 (0.01)	7.07 (0.47)
SHERM	0.80 (0.01)	0.67 (0.01)	0.72 (0.01)	0.90 (0.01)	7.03 (0.41)
*p*-value (U-Net vs. RATS)	<0.05	<0.05	N.S.	<0.001	N.S.
*p*-value (U-Net vs. PCNN)	<0.001	<0.001	N.S.	<0.001	<0.005
*p*-value (U-Net vs. SHERM)	<0.001	<0.001	N.S.	<0.001	<0.005

*The *p*-value indicates the result of two-tailed paired *t*-test comparison (best performance results in bold).*

**TABLE 3:** Quantitative comparison of U-Net, RATS, PCNN, and SHERM for segmentations on mouse T2^∗^w EPI images from Online dataset.

Methods	Dice	Jaccard	PPV	SEN	Hausdorff (voxels)
U-Net	**0.92 (0.01)**	**0.85 (0.01)**	0.91 (0.01)	**0.93 (0.01)**	**3.57 (0.14)**
RATS	0.85 (0.01)	0.75 (0.01)	**0.97 (0.00)**	0.76 (0.01)	3.85 (0.11)
PCNN	0.87 (0.01)	0.77 (0.01)	0.86 (0.01)	0.88 (0.01)	3.79 (0.16)
SHERM	0.87 (0.01)	0.77 (0.01)	0.92 (0.01)	0.82 (0.01)	3.39 (0.10)
*p*-value (U-Net vs. RATS)	<0.001	<0.001	<0.001	<0.001	N.S.
*p*-value (U-Net vs. PCNN)	<0.001	<0.001	<0.005	<0.001	N.S.
*p*-value (U-Net vs. SHERM)	<0.001	<0.001	N.S.	<0.001	N.S.

*The *p*-value indicates the result of two-tailed paired *t*-test comparison (best performance results in bold).*

## Discussion

Our results indicate that our proposed skull stripping framework based on U-Net represents a robust method for the accurate and automatic extraction of rodent brain tissue from MR images. While existing rodent skull stripping methods are robust when used with high-resolution anatomical images, most of them face challenges with low resolution, low contrast T2^∗^w EPI datasets. Overall, the U-Net based method showed consistent performance in both T2w RARE and T2^∗^w EPI, likely attributed to the use of both T2w RARE and T2^∗^w EPI images to train our U-Net architecture.

Compared to the pioneering techniques RATS (Oguz et al., 2014), PCNN (Chou et al., 2011), and SHERM (Liu et al., 2020), our proposed U-Net architecture is more robust, likely due to its capability to explore and learn the hierarchical features from the training dataset without requiring additional parameter adjustments. U-Net combines the location information from the downsampling path with the contextual information in the upsampling path to obtain a combination of localization and contextualization necessary to predict a reliable segmentation (Ronneberger et al., 2015). One clear advantage of the U-Net algorithm is that it is parameter free in the segmentation process, as all parameters are automatically learned from the data itself. The only parameters to learn on convolution layers in U-Net are the kernel. The size of the kernel is independent from the input image size, so images of different sizes can be used as input. In contrast, both RATS and PCNN need to select the appropriate brain size for rat or mouse brain for accurate justification. In RATS, the intensity threshold also needs to be adjusted to remove low signal intensity as potential non-brain signal. In practice, users need to adjust these parameters once per study based on the acquisition protocol, which affects the intensity profile, and the age/species/strain of the animals, which affects expected brain sizes. Note that, RATS, PCNN, and SHERM still reach an accurate (Dice > 0.8) and fast segmentation performance whereas the U-Net architecture requires longer processing time and needs a higher level of computational power for architecture training. Typically, deep learning-based methods are time-consuming in central processing units (CPU) but are significantly more time-efficient in graphics processing units (GPU). Indeed, the computation time of our proposed U-Net application can benefit significantly by using GPU (Supplementary Figure 4). Besides, conventional rodent brain extraction algorithms were based on prior knowledge of rodent brain anatomy, or adapting a general-purpose segmentation method, so an image covering the complete rat brain is necessary for basic functioning. In contrast, since the U-Net architecture learns the features for each slice, it could still work with images covering a limited brain section.

The robustness of U-Net is clearly illustrated in the segmentation performance of selected-cases across different protocols. Due to relatively poor signal intensity in the brainstem, olfactory bulb, and inferior part of brain, RATS, PCNN, and SHERM displayed lower segmentation accuracy in these areas in T2w RARE and T2^∗^w EPI. Although all methods provided outstanding segmentation performance (Dice > 0.9), the best T2w RARE and T2^∗^w EPI segmentation comparisons still showed mismatches in the inferior part of brain in RATS, PCNN, and SHERM. Furthermore, outcome assessments using different MRI protocols (T2w RARE and T2^∗^w EPI images) indicate that U-Net has high accuracy and consistency across various resolutions. Notably, while most brain segmentation was performed in the anatomical image (T2w RARE), our proposed U-Net architecture also shows accuracy in the T2^∗^w EPI images. When comparing the skull stripping results between T2w RARE and T2^∗^w EPI images in the CAMRI dataset, PCNN, RATS, and SHERM showed significantly lower segmentation accuracy in T2^∗^w EPI images while no significant difference was displayed in the U-Net algorithm. Specifically, in the worst case of T2w RARE image (Figure 3), the RATS displayed PPV = 0.99 and SEN = 0.79, which indicated the identified brain tissue has a high rate of true positive but low rate of false negative predictions, and the opposite performance was found in PCNN (PPV = 0.79 and SEN = 0.82). A similar trend was also found in the worst case of T2^∗^w EPI image (Figure 3). The T2^∗^w EPI outcome in RATS is underestimated and in PCNN is overestimated, which makes U-Net the superior choice for skull stripping these lower resolution images (PPV = 0.99 and SEN = 0.94). We observed the similar skull stripping performance for T2 W (Dice = 0.97) and EPI (Dice = 0.96), indicating that the model is adequately trained and not susceptible to ghosting artifacts in EPI. Rodent EPI data is also less prone to motion because the subjects are either under anesthesia and secured with ear and tooth bars (Atay et al., 2008; Albaugh et al., 2016; Van Den Berge et al., 2017; Broadwater et al., 2018; Grandjean et al., 2019, 2020; Sirmpilatze et al., 2019; Mandino et al., 2020) or awake and tightly restrained (Madularu et al., 2017; Ma et al., 2018). Indeed, none of the dataset available on online repository suffers from severe EPI ghosting.

To illustrate the reliability of our proposed U-Net architecture, we included independently generated rat and mouse public datasets (Online dataset), including images acquired from different sites, scanners, and protocols. The presented results showed that U-Net produced stable and satisfactory results for both T2w RARE and T2^∗^w EPI images. Although segmentation performance was not as robust in the mouse dataset, U-Net still reached significantly higher segmentation accuracy with averaged Dice > 0.85 for both T2w RARE and T2^∗^w EPI compared to other methods, whereas the lowest averaged accuracy on all images was found in RATS (Dice = 0.82), PCNN (Dice = 0.79), and SHERM (Dice = 0.80) for mouse T2w RARE. This result highlights the reliable performance of the U-Net architecture for mouse brain MRI data.

There are several limitations of the U-Net architecture. First, deep learning is a data driven classification, so segmentation accuracy highly relies on the training dataset. Indeed, we observed in Supplementary Figures S1, S2 that manual segmentation accuracy is approximately the same as validation accuracy. Because we trained our U-Net algorithm by using only T2w RARE and T2^∗^w EPI images in rats and mice, additional training and optimization will be needed to use our current U-Net architecture to skull-strip rodent brain images with different contrast (e.g., T1-weighted images). There are many challenges with conducting deep learning algorithm in multimodality datasets (i.e., heterogeneous sources, different levels of noise) (Ngiam et al., 2011; Baltrusaitis et al., 2019) as the features have to relate multiple data sources. Our future work will focus on developing rodent brain extraction tool specifically for T1w images. Second, deep learning methods require substantial amounts of manually labeled data (Verbraeken et al., 2020), and their performance can be affected by similarities between the training dataset and the unanalyzed dataset. The use of massive data augmentation is important in domains like biomedical segmentation, since the number of annotated samples is usually limited. More training datasets are needed to further improve our current U-Net architecture (e.g., including an additional mouse dataset with ground truth labels to improve our U-Net performance in mice). Third, our current U-Net architecture image patch limits the testing image to a matrix size of at least 128 × 128. Image resampling to a finer resolution is required if the image matrix size is smaller than 128 × 128. Fourth, whether 2D or 3D framework would yield better skull stripping or segmentation results remain an active topic of research (Baumgartner et al., 2018; Hänsch et al., 2018; Meine et al., 2018; Yu et al., 2019). Practically, each framework has its own advantages and disadvantages. For example, though 2D frameworks do not utilize information across slice direction and may only be suitable when slice resolution is coarse, they are also operationally efficient due to lower computational demands. Our results indeed support the feasibility of performing 2D U-Net framework in regular laptop CPU. The 3D framework, in contrast, preserves 3D context in training but suffers from inaccuracy when only limited number of slices is available. Finally, our future work will extend this study with more detailed classification of brain area labels so that automatic segmentation of brain nuclei using U-Net can be achieved.

## Conclusion

The robustness of U-Net for delineating rodent brain boundaries are demonstrated in T2w RARE and T2^∗^w EPI data acquired at multiple sites. Our proposed method demonstrated improved performance compared to current skull stripping methods, as determined using the qualitative metrics (Dice, Jaccard, PPV, SEN, and Hausdorff). We believe this tool will be useful to avoid parameter-selection bias and streamline pre-processing steps when analyzing rodent brain MRI data. Information about the CAMRI dataset used in this manuscript and our U-Net skull stripping tool can be found at https://github.com/CAMRIatUNC/RodentMRISkullStripping.

## Data Availability Statement

The datasets presented in this study can be found in online repositories. The CAMRI rats dataset is available at https://openneuro.org/datasets/ds002870/versions/1.0.0 and mice dataset is available at https://doi.org/10.18112/openneuro.ds002868.v1.0.0. The U-Net skull stripping tool can be found at https://github.com/CAMRIatUNC/RodentMRISkullStripping. The names of the repository/repositories and accession number(s) can be found in the article/Supplementary Material.

## Ethics Statement

Ethical review and approval was not required for the animal study because existing animal imaging database was used. No animal data acquired specifically for this project.

## Author Contributions

L-MH, DS, and Y-YS designed the study. L-MH and SW implemented U-Net algorithm for rodents. L-MH and PR validated the developed methods on various datasets. WB provided ground-truth brain masks. T-HC, SS, DC, LW, MB, and S-HL provided data and helped to edit the manuscript. S-HL managed data/software dissemination and helped to design the study. L-MH and Y-YS wrote the manuscript. All authors contributed to the article and approved the submitted version.

## Conflict of Interest

The authors declare that the research was conducted in the absence of any commercial or financial relationships that could be construed as a potential conflict of interest.

## Footnotes

1 https://doi.org/10.18112/openneuro.ds001981.v1.0.3

2 https://doi.org/10.18112/openneuro.ds001541.v1.1.2

3 https://doi.org/10.18112/openneuro.ds001720.v1.0.2

4 http://stnava.github.io/ANTs/
