# Supplementary material for: Automatic Skull Stripping of Rat and Mouse Brain MRI Data Using U-Net
Source: Front Neurosci. 2020 Oct 7;14:568614. doi: 10.3389/fnins.2020.568614 (PMC7575753; doi:10.3389/fnins.2020.568614)
Supplement: Supplementary file 1 [file Data_Sheet_1.docx]

Supporting information:

Figure S1. Performance validation results within the training process. In the training process, we further randomly selected 80% of the rat data from the training dataset (62 SD, 12 LE, and 10 Wistar) and included all mouse data for inner training. The remaining 20% of the rat data from the training dataset was used for validating the U-Net model. We repeated this training-validation process for five times to avoid randomness in data splitting. The U-Net model with highest averaged validation accuracy was then used as the final model for testing.

Figure S2. Accuracy of ground truth. Data from two anatomical experts were included (double-blinded). Compared to the ground truth used in this study, inter-rater accuracy was calculated on T2w RARE and T2*w EPI images using randomly selected 20 rats from the CAMRI dataset.
Figure S3. Segmentation performance for U-Net, RATS, PCNN, and SHERM on the T2*w EPI images from CAMRI dataset. Dice score averaged over the four methods (U-Net, RATS, PCNN, and SHERM).

Figure S4. Computation time for U-Net algorithm on the T2w RARE and T2*w EPI images from CAMRI dataset under CPU and GPU environment. The computation time was estimated on a Linux-based (Red Hat Enterprise Linux Server release 7.4 (Maipo)) computing system (Intel E5-2680 v3 processor, 2.50 GHz, 256-GB RAM) with CPU only and GPU (8 GeForce GTX1080 gpus, 8 physical cores, 2.60 GHz Intel processors, 10M cache (Model E5-2623 v4), 64 GB memory, 2x10Gbps NIC, 2560 NVIDIA CUDA cores, and 8 GB GDDR5X memory).
